# Diabetic nephropathy: recent advances in pathophysiology and challenges in dietary management

**DOI:** 10.1186/s13098-019-0403-4

**Published:** 2019-01-23

**Authors:** Mahaboob Khan Sulaiman

**Affiliations:** Fatima College of Health Sciences, PO Box 57788, Abu Dhabi, UAE

**Keywords:** Diabetic nephropathy, Chronic kidney disease, Dietary intervention, Proteinuria, Malnutrition

## Abstract

**Background:**

Diabetic nephropathy (DN) or diabetic kidney disease refers to the deterioration of kidney function seen in chronic type 1 and type 2 diabetes mellitus patients. The progression of the disease is known to occur in a series of stages and is linked to glycemic and blood pressure control. However, despite aggressive blood sugar control the prevalence of chronic kidney disease (CKD) in diabetic patients has not witnessed any decrease in the last two decades; which has lead to identification of additional factors in its progression. The nutritional status of patients is an important and modifiable factor that may influence CKD processes and outcome. It directly stems from the traditional dietary choices that patients make due to poor nutritional awareness. Dietary management of DN patients is challenging, as the twin factors of diet overload on kidney function needs to be balanced with malnutrition. Patient education seems to be the key in avoiding overindulgence of carbohydrate and protein-rich foods while favoring inclusion of essential fats in their diet.

**Conclusion:**

This review will summarize current advances in staging and molecular pathogenesis of DN. It will highlight recent studies focusing on patient-customized dietary interventions that offer new hope as an effective tool in improving quality of life and delaying disease progression in DN patients.

## Introduction

In 2015, the International Diabetic Federation estimated that the prevalence of diabetes was 8.8% from ages 20 to 79 years affecting a population of approximately 440 million people [1]. This is predicted to grow to over 550 million people by the year 2035 [2]. One of the most important clinical features of diabetes is its association with chronic tissue complications. A short-term increase in hyperglycemia does not result in serious clinical complications. The duration and severity of hyperglycemia is the major causative factor in initiating organ damage. Early morphological signs of renal damage include nephromegaly and a modified Doppler, but the degree of damage is best ascertained from proteinuria and Glomerular filtration rate (GFR) [3]. The average incidence of diabetic nephropathy is high (3% per year) during the first 10 to 20 years after diabetes onset [4]. Typically, it takes 15 years for small blood vessels in organs like kidney, eyes and nerves to get affected. It is estimated that more than 20 and up to 40% of diabetic patients will develop chronic kidney disease (CKD) [5, 6], depending upon the population, with a significant number that develop end stage kidney disease (ESKD) requiring renal replacement therapies such as kidney transplantation. Incidentally, diabetes with no clinical sign of kidney damage during the initial 20 to 25 years is significantly less likely (1% a year) to cause major renal complication later in life [4].

## Staging of diabetic nephropathy

Until recently, diabetic nephropathy was defined by the evidence of proteinuria ≥ 300 mg/day, in a diabetic patient [7]. Although urinary albumin is recognized as an early marker of DN, significant glomerular damage has already occurred when albumin appears in urine. Therefore, novel urinary biomarkers are needed to identify patients who are at risk of developing kidney damage. A proteomic study of the condition collectively termed as non-albumin proteinuria (NAP) identified several putative early biomarkers such as α-1 microglobulin, β-1 microglobulin, Nephrin, Cystatin C etc., [8]. While these markers can serve as sensitive early indicators of tubule damage, currently, they are neither calibrated nor universally available [9]. Moreover, precipitation of morning urine proteins and subsequent resolution by 2D electrophoresis also identified another putative urinary biomarker kininogen-1. This protein involved in the kallikrein-kinin system also awaits validation in larger cohorts [10].

Several recent studies have enabled a more robust and comprehensive stratification of DN. In 2010, Tervaert et al. reported a new pathological classification of kidney lesions that involved tubules, interstitium and/or the vessels as shown in Table 1 [11]. Such a classification was required, as a considerable percentage of patients with diabetes and impaired renal filtration do not exhibit elevated protein excretion. Also, many patients with Type 1 DM show proteinuria without concurrent GFR changes. Since diabetes mellitus studies are often observational and lack biopsy data to prove involvement of lesions, diabetic nephropathy is now classified as diabetic kidney disease (DKD). Interestingly, these classical stages of type 1 DM (T1DM) may not occur in type 2 DM (T2DM) patients as the latter is often diagnosed with concurrent disorders such as hypertension, proteinuria and renal failure [11, 12]. Therefore, a new term diabetic chronic kidney disease (DCKD) was proposed to replace diabetic nephropathy to explain the extent of kidney damage. Additionally, in these patients with type 2 DM, it is recommended that screening should be performed at diagnosis and yearly thereafter. More recently, Gheith et al. [13] have proposed five stages of diabetic nephropathy after a comprehensive review of literature as summarized in Table 1.

**Table 1:** Staging of diabetic nephropathy

Stages	DN staging Tervaert et al. [11]	DN staging Gheith et al. [13]
Stage 1	Glomerular basement membrane thickening	From onset to 5 years. Borderline GFR, no albuminuria, hypertension. But kidney size increased by 20% along with an increase in renal plasma flow
Stage 2	Mild or severe mesangial expansion	From 2 years after onset with basement membrane thickening and mesangial prolieration, normal GFR and no clinical symptoms
Stage 3	Nodular sclerosis	5–10 years after onset with or without hypertension, with glomerular damage and microalbuminuria (30–300 mg/day)
Stage 4	Advanced diabetic glomerulosclerosis that includes tubulointerstitial lesions and vascular lesions	Irreversible proteinuria, sustained hypertension and GFR below 60 ml/min/1.73 m^2^
Stage 5	–	End-stage kidney disease with GFR < 15 ml/min/1.73 m^2^

*DN* diabetic nephropathy, *GFR* glomerular filtration rate

## Risk factors for diabetic nephropathy

Many epidemiological studies demonstrate that ethnicity, family history, gestational diabetes, elevated blood pressure, dyslipidaemia, obesity and insulin resistance are the major risk factors of diabetic nephropathy [14]. Other putative risk factors include elevated glycosylated haemoglobin level (HbA1c), elevated systolic pressure, proteinuria and smoking [15].

## Modifiable vs non-modifiable risk factors: recent advances

Although nephropathy is the strongest predictor of mortality in patients with diabetes, its development involves important inter-individual variations. Genome-wide transcriptome studies [16] and high-throughput technologies [17] indicate the activation of inflammatory signaling pathways and oxidative stress highlighting the role of genetic factors. Evidences suggest that epigenetic mechanisms such as DNA methylation, noncoding RNAs and histone modifications can also play a pivotal role in the pathogenesis of diabetic nephropathy. Accordingly, cytokine TNF-alpha, IL-6 and IL-1 beta gene promoter polymorphisms and modulation in expression have been linked to DN susceptibility in subjects.

Dysregulation of local metabolic environment triggered by inflammation and subsequent tissue remodeling may initiate kidney damage [18]. Excess intracellular glucose have been shown to activate cellular signaling pathways such as diacylglycerol (DAG)-protein kinase C (PKC) pathway, advanced glycation end-products (AGE), polyol pathway, hexosamine pathway and oxidative stress [19]. Many studies have linked these pathways to key steps in the development of glomerulosclerosis. In addition to these metabolic pathways, Rho-kinase, an effector of small-GTPase binding protein Rho, has been linked to various steps in the ultra structural damage of diabetic nephropathy by inducing endothelial dysfunction, mesangial excessive extracellular matrix (ECM) production, podocyte abnormality, and tubulointerstitial fibrosis. A review on the important pathways that lead to diabetic nephropathy can be found elsewhere [20].

## Type of diabetes and their progression to diabetic nephropathy

Although microalbuminuria is a confirmatory test for diagnosis of diabetic nephropathy, not all patients progress to macroalbuminuria. In fact, some patients may regress to normoalbuminuria [21]. The progression of kidney disease in type 1 diabetes mellitus is unpredictable and seems to be connected to the intensity of blood sugar and pressure control. Accordingly, while initial studies reported that ~ 80% microalbuminuric patients progress to proteinuria over 6–14 years [22, 23], recent studies have reported a regression as a result of better glycemic control. For example, the Joslin type 1 cohort and DCCT/EDIC study reported roughly similar results of 58% patients and 50% patients with microalbuminuria regressed to normoalbuminuria over 6 years and within 10 years, with or without renin–angiotensin–aldosterone system (RAAS) inhibitors respectively, solely with better control of diabetes, hypertension and lipids [24, 25]. Improvement in microalbuminuria also resulted in 89% lower risk of developing a decreased GFR in type 1 DM patients.

In contrast, progression and regression of kidney disease in type 2 DM is highly variable as it is usually diagnosed with a secondary disorder, the onset of which is unrecorded. The UKPDS study reported microalbuminuria and reduced GFR in 38% and 29% patients respectively after a median follow-up of 15 years [26]. In terms of progression, the same study reported a change from microalbuminurea–macroalbuminuria-ESKD at 2.8% and 2.3% per year respectively. In contrast, the Pima Indians study reported that macroalbuminuria was 50% during a median follow-up of 20 years [27]. Also, a gradual loss of kidney damage with time was noticed as 7.3% patients were diagnosed with microalbuminuria at the onset, 17.3% at 5 years, 24.9% at 10, and 28% at 15 years. Epidemiological studies in Western and Pima Indian populations also suggest that the prevalence of overt nephropathy is about 21% in patients with type 1 DM, and 20–25% in patients with Type 2 DM, depending solely on the duration since onset of disease.

## Potential serum biomarkers of diabetic nephropathy: recent advances

Traditionally, biomarkers are evaluated based on their ability to predict the onset or monitor the progression of DN. As albuminuria has certain limitations the quest for more reliable serum and renal biomarkers with higher sensitivity and specificity has led to an explosion of literature in this field. MacIssac et al. [28] have presented a detailed review of current literature on relevant biomarkers. Recently, Motawi et al. [29] estimated three new promising biomarkers: neutrophil gelatinase-associated lipocalin (NGAL), beta-trace protein (beta TP) and microRNA-130b (miR-130b) in type 2 DM. They concluded that serum NGAL and betaTP were significantly elevated in T2DM patients and can serve as early biomarkers of tubular and glomerular markers respectively. Other recent reviews on the promise of biomarkers in early detection of DKD can also be seen [30]. Such advances in biomarker research and metabolic phenotyping offer hope for multiparametric risk assessment of kidney injury and effective interventional strategies in future.

## Diet therapy in diabetic nephropathy and its importance

The primary goal of diabetic nephropathy treatment is to prevent microalbuminuria from progressing to macroalbuminuria and an eventual decrease in renal function and associated heart disorders. Consequently, intensive glycaemic control, antihypertensive treatment by blocking RAAS system and lipid-modifying statin therapy are the main cornerstones of treatment. A detailed discussion of the various treatment methods of diabetic nephropathy is beyond the scope of this article, and reviews on the subject are available [31–33].

The nutritional status of patients is an important and modifiable factor that may influence DN processes and outcome [34]. Diet is a crucial factor in influencing the nutritional status of an individual. Whereas diabetes advocates a healthy and balanced diet, diet of a CKD or diabetic nephropathy patient is challenging and designed to delay progression of kidney damage and the associated secondary conditions such as hypertension, hyperlipidemia, uremia, etc. It also needs continuous monitoring and must be personalized to the patients’ treatment regimen. As food intake could be a burden on kidney function, a delicate balance between nutrition and sustainable physiological load is essential to maintain quality of life for the patient. A common problem encountered in patients with renal failure and proteinuria is their lack of nutritional knowledge and continued adherence to traditional food choices that are rich in carbohydrate, proteins or minerals. Since a majority of patients are dyslipidemic the only control they exercise is on limiting fat intake. Such a skewed diet places a tremendous burden on kidney function that causes further problems in disease management.

An ideal diet recommended for diabetic nephropathy patients with compromised kidney function includes a proper amount of fat to prevent malnutrition. More so when total calories coming from protein and carbohydrate intake needs to be restricted. A total fat reduction as advised by earlier studies can be a very unhealthy practice. Thus, to achieve these goals nutritionists advice limiting saturated fatty acid consumption while taking vegetable oils and omega-rich fatty acid containing oils in moderation. Many clinical studies have highlighted the renoprotective effects of a low protein diet on DN, although protein restriction alone does not result in a positive outcome for patients [35]. Moreover, a protein-deficient diet (0.6 to 0.7 g/kg/day) needs to be integrated into the overall care of renal insufficiency with customized dietary interventions to avoid malnutrition [36]. Interestingly, in animal type 2 DM models a very low protein diet (VLPD) improved tubulo-interstitial damage, inflammation and fibrosis, through restoration of autophagy via reduction of a mammalian target of rapamycin complex 1 (mTORC1) activity [37]. Although a low protein diet slows progression of renal dysfunction in human subjects with chronic glomerular nephritis, VLPD has not been clinically validated. A low-salt diet that is devoid of salted and pickled foods is highly recommended for DN patients. Restricted sodium intake allows better blood pressure control in such patients. High salt intake and urinary protein excretion were associated with annual creatinine clearance decline in type 2 DKD patients as reported by Kanauchi et al. [38]. Potassium is an essential electrolyte involved in the contraction and relaxation of muscles. During a deficit in kidney function potassium excretion is reduced leading to an accumulation in body tissues. Therefore, potassium intake specifically from foods such as grains, potatoes, corn, soybean, nuts, tomatoes, banana, melons, kiwi etc. must be restricted. Like potassium, phosphorus excretion is also reduced during chronic kidney damage leading to increased blood phosphorus levels. Since phosphate is in homeostatic equilibrium with the skeletal muscle calcium levels, an imbalance leads to a significant calcium loss and debilitating bone disease. In summary, excessive carbohydrate and protein intake is managed with a target of 1600 kcal of energy per day in which 60 percent comes from carbohydrate and 40 percent from proteins. In a recent study, such a regimen achieved a commendable control in blood lipid and glucose values in a patient with stage 4 chronic kidney disease [39]. However, patient adherence to the recommended diet seems to be gender-specific. For example, Ahola et al. [40] assessed frequency of adherence to special diet in a large cohort Finnish DN study and reported that adherents were more frequently women, older, and had longer duration of diabetes. Therefore, effective adherence through patient education may be a crucial factor in the management of DN through diet.

In conclusion, this review summarizes the recent advances in the pathophysiology of diabetic nephropathy and the importance of dietary factors in modifying treatment outcomes for patients. A critical analysis of studies that emphasize the importance of patient-centered dietary intervention in successful management of advanced CKD patients has been presented. Large-scale cohort studies are necessary to evaluate the efficiency of diet as a new therapeutic paradigm. Nevertheless, proactive personalized diet-management plans tailored to the disease stage is likely to be the future trend in diabetic nephropathy therapy as it will have a large impact on the patient’s quality of life and may prolong survival. Notably, in newly diagnosed DN patients these dietary interventions may no longer be regarded as complementary measures but significant factors that delay progression of the disease.

## Abbreviations

CKD: chronic kidney disease
DN: diabetic nephropathy
GFR: glomerular filtration rate
ESKD: end stage kidney disease
DM: diabetes mellitus
DKD: diabetic kidney disease
